# 4,4′-Bi­pyridine-1,1′-diium tetra­chloridodi­fluorido­stannate(IV) monohydrate

**DOI:** 10.1107/S2414314625005966

**Published:** 2025-07-11

**Authors:** Amina Kemmouche, Rochdi Ghallab, Hocine Merazig

**Affiliations:** a Ecole Nationale Superieure de Biotechnologie de Constantine, Algeria; b Laboratoire de Technologie des Materiaux Avances Ecole Nationale Polytechnique de Constantine Algeria; c Unite De Recherche Chimie De L'Environnement Et Moleculaire Structurale URCHEMS University of Constantine 1-Mentouri Brothers Algeria; University of Aberdeen, United Kingdom

**Keywords:** crystal structure, stannate(IV), bipyridinium, X-ray diffraction

## Abstract

In the title hydrated salt, (C_10_H_10_N_2_)[SnF_2_Cl_4_]·H_2_O, the dihedral angle between the pyridinium rings in the cation is 40.5 (4)° and the F atoms in the octa­hedral complex anion have a *cis* disposition [F—Sn—F = 85.32 (17)°]. In the extended structure, alternating cationic and anionic layers occur, linked by extensive hydrogen bonding, with water mol­ecules inserted between the layers.

## Abstract 2

The F atoms in the octa­hedral [SnF_2_Cl_4_]^2–^ complex anion of the title compound are in a *cis* orientation.

## Graphical abstract

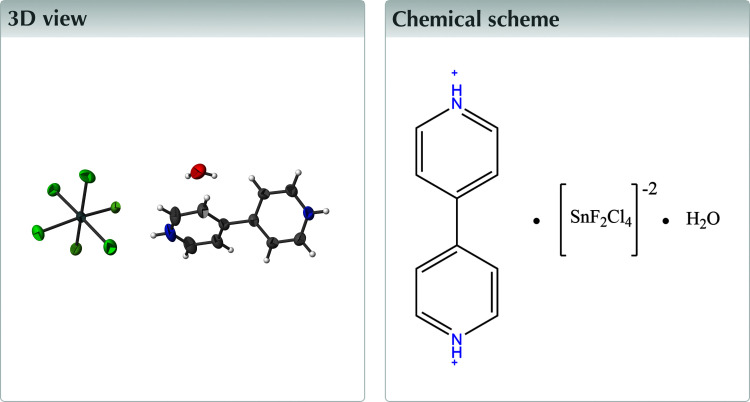

## Structure description

**Figure 1:**
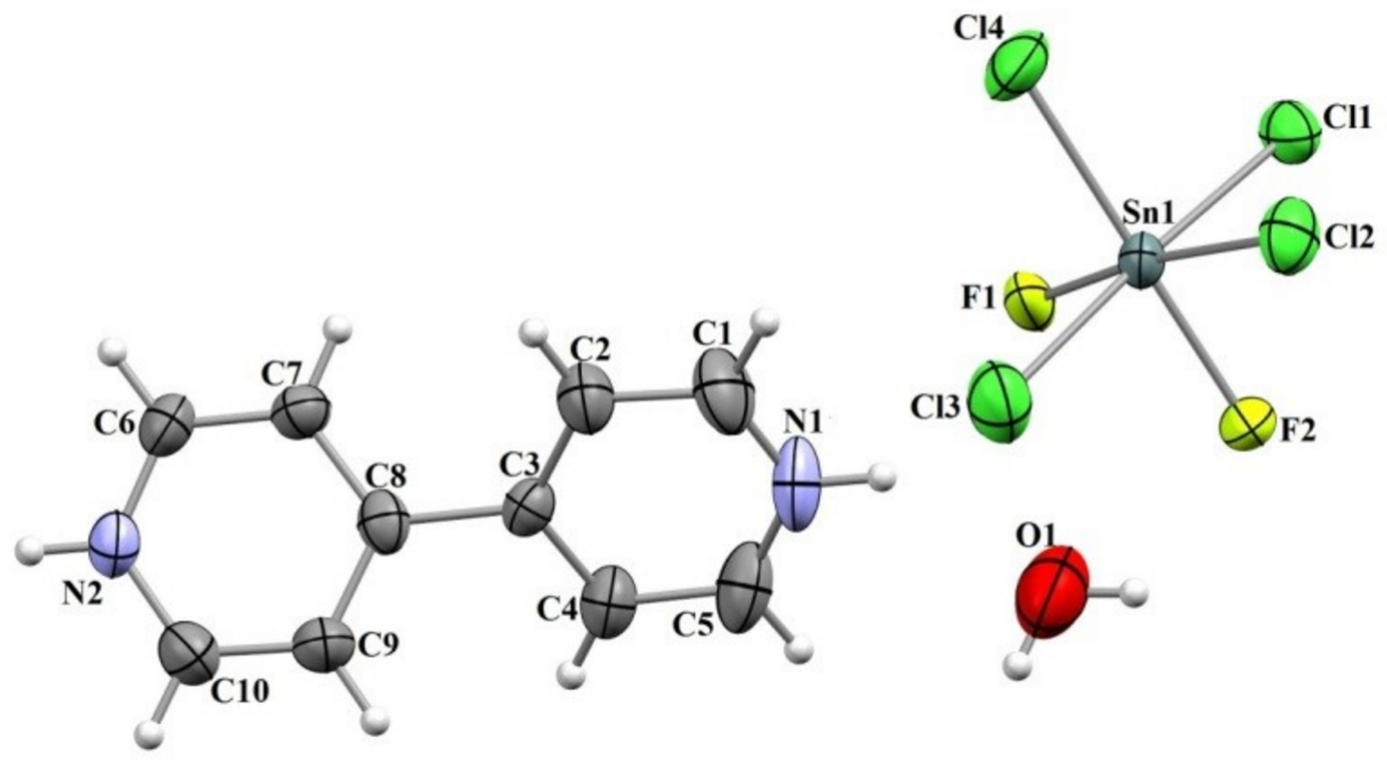
The mol­ecular structure of the title compound with displacement ellipsoids drawn at the 50% probability level.

The title compound, (C_10_H_10_N_2_^2+^)[SnF_2_Cl_4_]^2–^·H_2_O, crystallizes in the non-centrosymmetric ortho­rhom­bic space group *Pna*2_1_ with one cation, one anion and one water mol­ecule in the asymmetric unit (Fig. 1 ▸). The bipyridinium cation exhibits structural parameters indicative of protonation: the inter-ring C—C bond length is 1.476 (7) Å, while the average intra-ring C—C bond length is 1.384 (3) Å, reflecting aromatic conjugation. The C—N bonds measure 1.341 (9) Å or less, which are shorter than those in neutral bi­pyridine, due to the cationic charge. Angular distortions are observed with C—C—C angles in the range 117.9 (5)°–121.0 (6)° and C—N—C angles of 122.4 (5) and 123.0 (6)°, in agreement with literature data (*e.g.*, Horiacha *et al.*, 2022 ▸). The dihedral angle between the C1–C5/N1 and C6–C10/N2 pyridinium rings is 40.5 (4)°, which can be attributed to intra­molecular (steric) inter­actions (Gheribi *et al.*, 2022 ▸). The [SnF_2_Cl_4_]^2–^ anion adopts a distorted octa­hedral coordination sphere around the Sn^IV^ atom, with Sn—Cl bond lengths ranging from 2.383 (2) to 2.418 (2) Å and Sn—F bonds of 2.030 (4) and 2.096 (4) Å. The bond angles deviate somewhat from ideal octa­hedral values, with notable examples being 87.98 (13)° for F1—Sn1—F2 and 93.84 (8)° for Cl2—Sn1—Cl4, consistent with previous reports (Bruhn & Preetz, 1996 ▸).

**Figure 2:**
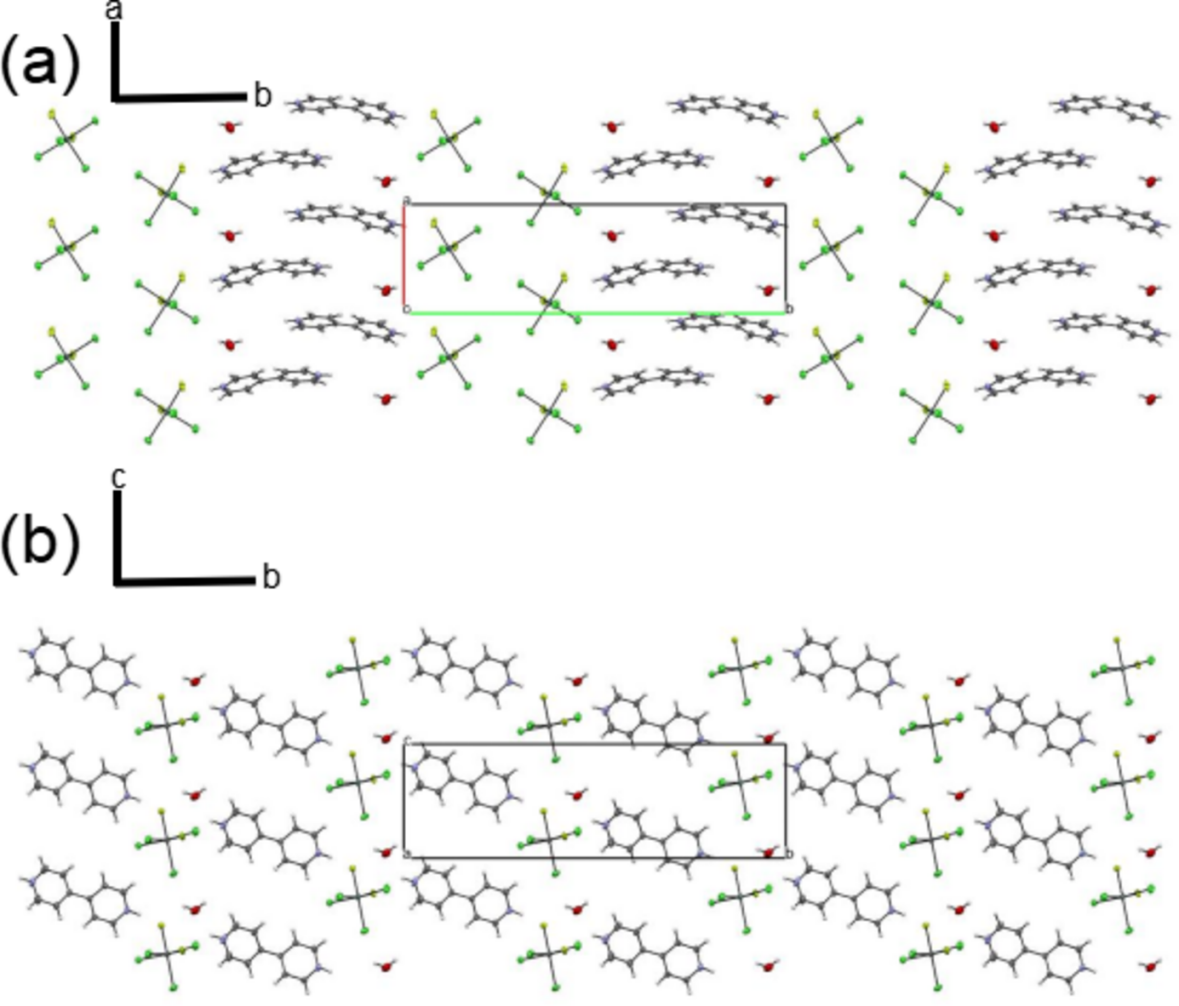
Projection of the crystal packing on (*a*) the *ab* plane and (*b*) the *bc* plane.

**Figure 3:**
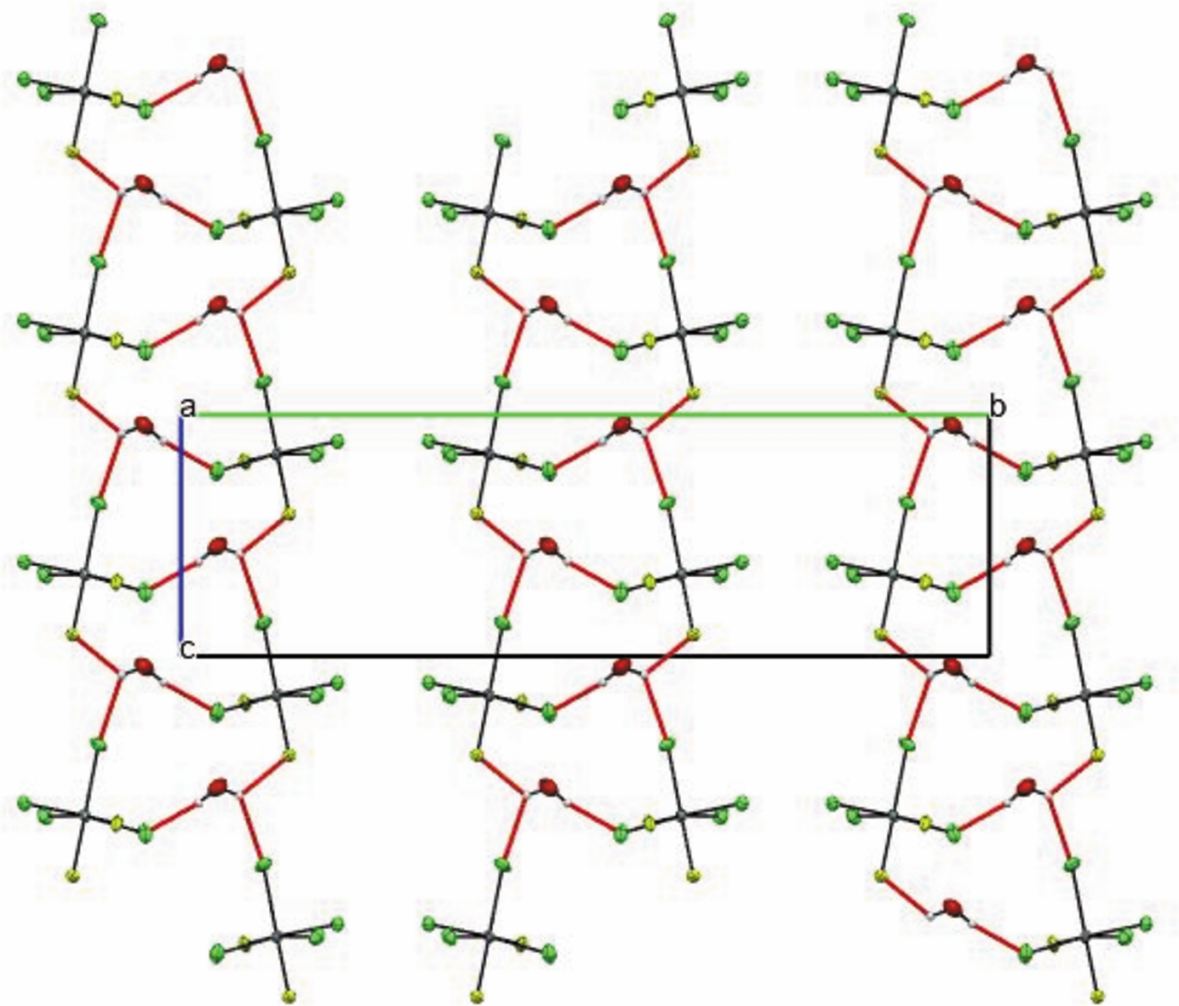
Sequence of 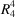
(12) loops in the structure.

The extended structure reveals alternating layers of cations and anions separated by inter­stitial water mol­ecules (Fig. 2 ▸). Eight hydrogen bonds consolidate the tri-periodic network (Table 1 ▸), including one strong (N1—H1⋯F2), two moderate (N2—H2⋯O1*W* and O1*W*—H1*WA*⋯Cl1), and five weak inter­actions (O1*W*—H1*WB*⋯Cl4, O1*W*—H1*WB*⋯F2, N2—H2⋯F1, C1—H1*A*⋯Cl3 and C9—H9⋯F1). Water mol­ecules mediate cyclic motifs in the *bc* plane *via* O1*W*—H⋯Cl/F inter­actions, generating a supra­molecular 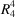
(12) graph-set motif (Fig. 3 ▸). Intra­layer cohesion is ensured by weak π–π stacking inter­actions with centroid–centroid distances of 3.950 (4) Å, while inter­layer bridging is provided by a Sn—Cl4⋯*Cg*(2 − *x*, 1 − *y*, −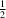 + *z*) halogen⋯π contact [Cl⋯π = 3.472 (4) Å, Sn—Cl⋯π = 110.42 (9)°] linking the [SnF_2_Cl_4_]^2–^ anion to the N2 ring of the bipyridinium cation. The crystal packing is thus supported by directional hydrogen bonds, face-to-face π-stacking within cationic layers, and anion–cation halogen⋯π contacts.

## Synthesis and crystallization

Tin(II) fluoride (1.56 mmol) was combined with 4,4′-bi­pyridine (1.56 mmol) in a 1:1 molar ratio. A few drops of hydro­chloric acid were added to the mixture in a minimal volume of distilled water to facilitate dissolution. After thorough stirring, the solution was transferred into a Biotage microwave vial (2–5 ml) and heated in an oven at 393 K for three days. Upon gradual cooling to room temperature, prismatic crystals of the title compound formed and were isolated under an optical microscope for further analysis.

## Refinement

Crystal data, data collection and structure refinement details are summarized in Table 2 ▸. The crystal studied was refined as a two-component inversion twin.

## Supplementary Material

Crystal structure: contains datablock(s) I. DOI: 10.1107/S2414314625005966/hb4521sup1.cif

Structure factors: contains datablock(s) I. DOI: 10.1107/S2414314625005966/hb4521Isup2.hkl

CCDC reference: 1904712

Additional supporting information:  crystallographic information; 3D view; checkCIF report

## Figures and Tables

**Table 1:** Hydrogen-bond geometry (Å, °)

*D*—H⋯*A*	*D*—H	H⋯*A*	*D*⋯*A*	*D*—H⋯*A*
N1—H1⋯F2^i^	0.86	1.79	2.629 (6)	163
N2—H2⋯F1	0.86	2.32	2.989 (6)	134
N2—H2⋯O1*W*^ii^	0.86	2.11	2.819 (10)	140
O1*W*—H1*WA*⋯Cl1^iii^	0.85	2.51	3.324 (8)	159
O1*W*—H1*WB*⋯Cl4^ii^	0.85	2.72	3.366 (8)	134
O1*W*—H1*WB*⋯F2^iv^	0.85	2.32	2.906 (9)	126
C1—H1*A*⋯Cl3^iv^	0.93	2.82	3.473 (6)	128
C9—H9⋯F1^ii^	0.93	2.44	3.109 (9)	129

Symmetry codes: (i) 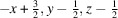; (ii) 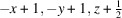; (iii) 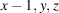; (iv) 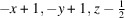.

**Table 2:** Experimental details

Crystal data	
Chemical formula	(C_10_H_10_N_2_)[SnCl_4_F_2_]·H_2_O
*M*_r_	474.71
Crystal system, space group	Orthorhombic, *Pna*2_1_
Temperature (K)	293
*a*, *b*, *c* (Å)	7.5641 (2), 26.5989 (5), 7.9422 (2)
*V* (Å^3^)	1597.94 (7)
*Z*	4
Radiation type	Mo *K*α
μ (mm^−1^)	2.28
Crystal size (mm)	0.08 × 0.08 × 0.07

Data collection	
Diffractometer	Bruker SMART APEXII area detector
Absorption correction	Multi-scan (*SADABS*; Krause *et al.*, 2015 ▸)
*T*_min_, *T*_max_	0.852, 0.852
No. of measured, independent and observed [*I* > 2σ(*I*)] reflections	16855, 7535, 6299
*R*_int_	0.030
(sin θ/λ)_max_ (Å^−1^)	0.837

Refinement	
*R*[*F*^2^ > 2σ(*F*^2^)], *wR*(*F*^2^), *S*	0.047, 0.107, 1.04
No. of reflections	7535
No. of parameters	182
No. of restraints	1
H-atom treatment	H-atom parameters constrained
Δρ_max_, Δρ_min_ (e Å^−3^)	1.11, −2.00
Absolute structure	Ad
Absolute structure parameter	0.06 (4)

Computer programs: *APEX2* and *SAINT* (Bruker, 2014 ▸), *OLEX2.solve* (Bourhis *et al.*, 2015 ▸), *SHELXL2019/3* (Sheldrick, 2015 ▸) and *OLEX2* (Dolomanov *et al.*, 2009 ▸).

## full crystallographic data

### 4,4'-Bipyridine-1,1'-diium tetrachloridodifluoridostannate(IV) monohydrate Crystal data

**Table:**

(C_10_H_10_N_2_)[SnCl_4_F_2_]·H_2_O	*D*_x_ = 1.973 Mg m^−^^3^
*M**_r_* = 474.71	Mo *K*α radiation, λ = 0.71073 Å
Orthorhombic, *P**n**a*2_1_	Cell parameters from 6299 reflections
*a* = 7.5641 (2) Å	θ = 1.5–36.5°
*b* = 26.5989 (5) Å	µ = 2.28 mm^−^^1^
*c* = 7.9422 (2) Å	*T* = 293 K
*V* = 1597.94 (7) Å^3^	Prism, colourless
*Z* = 4	0.08 × 0.08 × 0.07 mm
*F*(000) = 920	

### 4,4'-Bipyridine-1,1'-diium tetrachloridodifluoridostannate(IV) monohydrate Data collection

**Table:**

Bruker SMART APEXII area detector diffractometer	6299 reflections with *I* > 2σ(*I*)
ω and φ scans	*R*_int_ = 0.030
Absorption correction: multi-scan (SADABS; Krause *et al.*, 2015)	θ_max_ = 36.5°, θ_min_ = 1.5°
*T*_min_ = 0.852, *T*_max_ = 0.852	*h* = −11→12
16855 measured reflections	*k* = −44→20
7535 independent reflections	*l* = −13→13

### 4,4'-Bipyridine-1,1'-diium tetrachloridodifluoridostannate(IV) monohydrate Refinement

**Table:**

Refinement on *F*^2^	Hydrogen site location: mixed
Least-squares matrix: full	H-atom parameters constrained
*R*[*F*^2^ > 2σ(*F*^2^)] = 0.047	*w* = 1/[σ^2^(*F*_o_^2^) + (0.0043*P*)^2^ + 2.4611*P*] where *P* = (*F*_o_^2^ + 2*F*_c_^2^)/3
*wR*(*F*^2^) = 0.107	(Δ/σ)_max_ = 0.003
*S* = 1.04	Δρ_max_ = 1.11 e Å^−^^3^
7535 reflections	Δρ_min_ = −2.00 e Å^−^^3^
182 parameters	Absolute structure: ad
1 restraint	Absolute structure parameter: 0.06 (4)
Primary atom site location: dual	

### 4,4'-Bipyridine-1,1'-diium tetrachloridodifluoridostannate(IV) monohydrate Special details

**Table:**

Geometry. All esds (except the esd in the dihedral angle between two l.s. planes) are estimated using the full covariance matrix. The cell esds are taken into account individually in the estimation of esds in distances, angles and torsion angles; correlations between esds in cell parameters are only used when they are defined by crystal symmetry. An approximate (isotropic) treatment of cell esds is used for estimating esds involving l.s. planes.
Refinement. Refined as a 2-component inversion twin. Hydrogen atoms on the aromatic rings were geometrically positioned and refined as riding atoms with *U*_iso_(H) = 1.2*U*_eq_(C).

### 4,4'-Bipyridine-1,1'-diium tetrachloridodifluoridostannate(IV) monohydrate Fractional atomic coordinates and isotropic or equivalent isotropic displacement parameters (Å^2^)

**Table:**

	*x*	*y*	*z*	*U*_iso_*/*U*_eq_	
N1	0.5711 (7)	0.22347 (18)	0.5432 (7)	0.0394 (10)	
H1	0.567363	0.192734	0.510167	0.047*	
N2	0.6786 (8)	0.4709 (2)	0.7922 (10)	0.0567 (16)	
H2	0.697543	0.501502	0.822841	0.068*	
C1	0.5305 (8)	0.2597 (2)	0.4340 (8)	0.0389 (11)	
H1A	0.495469	0.251527	0.325148	0.047*	
C2	0.5406 (8)	0.3090 (2)	0.4830 (7)	0.0355 (10)	
H2A	0.511969	0.334401	0.407206	0.043*	
C3	0.5935 (6)	0.32135 (16)	0.6459 (10)	0.0321 (10)	
C4	0.6285 (8)	0.2819 (2)	0.7580 (8)	0.0383 (11)	
H4	0.659405	0.288740	0.868982	0.046*	
C5	0.6171 (9)	0.2333 (2)	0.7030 (8)	0.0437 (13)	
H5	0.641172	0.206937	0.776600	0.052*	
C6	0.6192 (7)	0.37422 (19)	0.6967 (7)	0.0337 (11)	
C7	0.6957 (9)	0.4080 (2)	0.5833 (9)	0.0449 (13)	
H7	0.724699	0.398189	0.474367	0.054*	
C8	0.7270 (10)	0.4569 (2)	0.6390 (15)	0.061 (2)	
H8	0.782381	0.479812	0.568010	0.073*	
C9	0.6021 (9)	0.4396 (3)	0.9022 (11)	0.0561 (19)	
H9	0.569590	0.451008	1.008552	0.067*	
C10	0.5717 (9)	0.3902 (2)	0.8562 (10)	0.0440 (13)	
H10	0.519959	0.367892	0.931787	0.053*	
Sn1	0.90313 (4)	0.61887 (2)	0.66064 (6)	0.03161 (8)	
Cl1	1.0622 (3)	0.54461 (7)	0.7330 (3)	0.0588 (5)	
Cl2	1.16953 (18)	0.66810 (5)	0.6621 (3)	0.0487 (3)	
Cl3	0.7297 (2)	0.69369 (5)	0.6093 (2)	0.0436 (3)	
Cl4	0.9182 (3)	0.60193 (8)	0.3643 (2)	0.0545 (4)	
F1	0.6661 (6)	0.57916 (12)	0.6921 (6)	0.0594 (12)	
F2	0.8830 (6)	0.63386 (14)	0.9106 (5)	0.0449 (8)	
O1W	0.2936 (11)	0.4547 (3)	0.5435 (9)	0.081 (2)	
H1WA	0.259704	0.478305	0.608329	0.122*	
H1WB	0.250934	0.426975	0.577499	0.122*	

### 4,4'-Bipyridine-1,1'-diium tetrachloridodifluoridostannate(IV) monohydrate Atomic displacement parameters (Å^2^)

**Table:**

	*U*^11^	*U*^22^	*U*^33^	*U*^12^	*U*^13^	*U*^23^
N1	0.045 (3)	0.028 (2)	0.045 (3)	0.0004 (17)	−0.001 (2)	−0.0094 (18)
N2	0.052 (3)	0.037 (3)	0.081 (5)	−0.008 (2)	0.002 (3)	−0.024 (3)
C1	0.044 (3)	0.037 (3)	0.036 (3)	−0.001 (2)	−0.002 (2)	−0.009 (2)
C2	0.040 (2)	0.034 (2)	0.032 (2)	0.001 (2)	−0.005 (2)	−0.0021 (19)
C3	0.0323 (17)	0.0280 (16)	0.036 (3)	−0.0013 (14)	0.005 (2)	−0.005 (2)
C4	0.046 (3)	0.035 (2)	0.034 (3)	0.001 (2)	−0.005 (2)	−0.001 (2)
C5	0.055 (3)	0.033 (2)	0.043 (4)	0.001 (2)	−0.001 (2)	0.003 (2)
C6	0.033 (2)	0.031 (2)	0.037 (3)	−0.0004 (15)	−0.0019 (17)	−0.0056 (17)
C7	0.050 (3)	0.033 (2)	0.052 (4)	−0.006 (2)	0.003 (3)	−0.005 (2)
C8	0.059 (4)	0.039 (3)	0.085 (7)	−0.018 (3)	0.011 (4)	−0.003 (4)
C9	0.052 (4)	0.049 (4)	0.068 (5)	−0.001 (3)	0.009 (3)	−0.028 (3)
C10	0.047 (3)	0.037 (3)	0.049 (3)	−0.004 (2)	0.001 (3)	−0.013 (2)
Sn1	0.03949 (14)	0.02310 (11)	0.03226 (13)	−0.00219 (10)	−0.00508 (19)	−0.00433 (15)
Cl1	0.0618 (10)	0.0384 (7)	0.0760 (12)	0.0042 (7)	−0.0140 (8)	−0.0060 (7)
Cl2	0.0445 (6)	0.0452 (6)	0.0565 (8)	−0.0130 (5)	0.0003 (10)	−0.0090 (10)
Cl3	0.0535 (8)	0.0351 (6)	0.0422 (7)	0.0112 (5)	0.0033 (6)	−0.0005 (5)
Cl4	0.0669 (10)	0.0578 (9)	0.0387 (8)	0.0074 (8)	−0.0084 (7)	−0.0199 (7)
F1	0.095 (3)	0.0278 (13)	0.055 (3)	−0.0150 (16)	−0.033 (2)	0.0077 (15)
F2	0.071 (2)	0.0327 (15)	0.0306 (16)	−0.0019 (16)	0.0021 (16)	−0.0019 (13)
O1W	0.102 (5)	0.074 (4)	0.067 (4)	−0.022 (4)	−0.009 (4)	0.025 (3)

### 4,4'-Bipyridine-1,1'-diium tetrachloridodifluoridostannate(IV) monohydrate Geometric parameters (Å, º)

**Table:**

N1—H1	0.8593	C6—C10	1.384 (9)
N1—C1	1.332 (8)	C7—H7	0.9300
N1—C5	1.341 (9)	C7—C8	1.394 (9)
N2—H2	0.8611	C8—H8	0.9300
N2—C8	1.325 (13)	C9—H9	0.9300
N2—C9	1.338 (12)	C9—C10	1.385 (9)
C1—H1A	0.9300	C10—H10	0.9300
C1—C2	1.372 (8)	Sn1—Cl1	2.3831 (18)
C2—H2A	0.9300	Sn1—Cl2	2.4032 (12)
C2—C3	1.393 (9)	Sn1—Cl3	2.4183 (14)
C3—C4	1.401 (8)	Sn1—Cl4	2.3994 (17)
C3—C6	1.476 (7)	Sn1—F1	2.096 (4)
C4—H4	0.9300	Sn1—F2	2.030 (4)
C4—C5	1.368 (9)	O1W—H1WA	0.8512
C5—H5	0.9300	O1W—H1WB	0.8498
C6—C7	1.397 (9)		
			
C1—N1—H1	118.8	C8—C7—H7	121.1
C1—N1—C5	122.4 (5)	N2—C8—C7	120.5 (7)
C5—N1—H1	118.8	N2—C8—H8	119.8
C8—N2—H2	118.7	C7—C8—H8	119.8
C8—N2—C9	123.0 (6)	N2—C9—H9	120.3
C9—N2—H2	118.3	N2—C9—C10	119.3 (7)
N1—C1—H1A	120.2	C10—C9—H9	120.3
N1—C1—C2	119.6 (5)	C6—C10—C9	119.3 (7)
C2—C1—H1A	120.2	C6—C10—H10	120.3
C1—C2—H2A	119.8	C9—C10—H10	120.3
C1—C2—C3	120.3 (5)	Cl1—Sn1—Cl2	91.56 (6)
C3—C2—H2A	119.8	Cl1—Sn1—Cl3	175.29 (7)
C2—C3—C4	117.9 (5)	Cl1—Sn1—Cl4	93.27 (7)
C2—C3—C6	121.0 (5)	Cl2—Sn1—Cl3	90.42 (5)
C4—C3—C6	121.0 (6)	Cl4—Sn1—Cl2	93.84 (8)
C3—C4—H4	120.2	Cl4—Sn1—Cl3	90.87 (6)
C5—C4—C3	119.6 (6)	F1—Sn1—Cl1	89.16 (12)
C5—C4—H4	120.2	F1—Sn1—Cl2	172.46 (14)
N1—C5—C4	120.1 (6)	F1—Sn1—Cl3	88.32 (12)
N1—C5—H5	119.9	F1—Sn1—Cl4	93.60 (14)
C4—C5—H5	119.9	F2—Sn1—Cl1	87.98 (13)
C7—C6—C3	119.4 (5)	F2—Sn1—Cl2	87.21 (13)
C10—C6—C3	120.5 (6)	F2—Sn1—Cl3	87.85 (12)
C10—C6—C7	120.0 (5)	F2—Sn1—Cl4	178.34 (13)
C6—C7—H7	121.1	F2—Sn1—F1	85.32 (17)
C8—C7—C6	117.8 (7)	H1WA—O1W—H1WB	109.4
			
N1—C1—C2—C3	0.2 (9)	C3—C6—C10—C9	178.1 (6)
N2—C9—C10—C6	−0.8 (11)	C4—C3—C6—C7	138.5 (6)
C1—N1—C5—C4	−1.9 (10)	C4—C3—C6—C10	−40.1 (8)
C1—C2—C3—C4	−2.5 (8)	C5—N1—C1—C2	2.1 (9)
C1—C2—C3—C6	174.9 (5)	C6—C3—C4—C5	−174.8 (5)
C2—C3—C4—C5	2.7 (8)	C6—C7—C8—N2	−2.7 (11)
C2—C3—C6—C7	−38.8 (7)	C7—C6—C10—C9	−0.5 (10)
C2—C3—C6—C10	142.5 (6)	C8—N2—C9—C10	0.2 (12)
C3—C4—C5—N1	−0.6 (9)	C9—N2—C8—C7	1.6 (13)
C3—C6—C7—C8	−176.4 (6)	C10—C6—C7—C8	2.2 (9)

### 4,4'-Bipyridine-1,1'-diium tetrachloridodifluoridostannate(IV) monohydrate Hydrogen-bond geometry (Å, º)

**Table:**

*D*—H···*A*	*D*—H	H···*A*	*D*···*A*	*D*—H···*A*
N1—H1···F2^i^	0.86	1.79	2.629 (6)	163
N2—H2···F1	0.86	2.32	2.989 (6)	134
N2—H2···O1*W*^ii^	0.86	2.11	2.819 (10)	140
O1*W*—H1*WA*···Cl1^iii^	0.85	2.51	3.324 (8)	159
O1*W*—H1*WB*···Cl4^ii^	0.85	2.72	3.366 (8)	134
O1*W*—H1*WB*···F2^iv^	0.85	2.32	2.906 (9)	126
C1—H1*A*···Cl3^iv^	0.93	2.82	3.473 (6)	128
C9—H9···F1^ii^	0.93	2.44	3.109 (9)	129

Symmetry codes: (i) −*x*+3/2, *y*−1/2, *z*−1/2; (ii) −*x*+1, −*y*+1, *z*+1/2; (iii) *x*−1, *y*, *z*; (iv) −*x*+1, −*y*+1, *z*−1/2.
